# Notch1 regulates endothelial apoptosis via the ERK pathway in chronic obstructive pulmonary disease

**DOI:** 10.1152/ajpcell.00182.2017

**Published:** 2018-06-06

**Authors:** Dandan Zong, Jinhua Li, Shan Cai, Shengdong He, Qingqing Liu, Jiehan Jiang, Shanshan Chen, Yingjiao Long, Yan Chen, Ping Chen, Ruoyun Ouyang

**Affiliations:** ^1^Department of Respiratory Medicine, the Second Xiangya Hospital, Research Unit of Respiratory Disease, Diagnosis and Treatment Center of Respiratory Disease, Central South University, Changsha, China; ^2^Department of Respiratory Medicine, Affiliated Hospital of Luzhou Medical College, Luzhou, China; ^3^Department of Respiratory Medicine, Changsha Central Hospital, Changsha, China; ^4^Department of Radiology, the Second Xiangya Hospital, Central South University, Changsha, China

**Keywords:** apoptosis, chronic obstructive pulmonary disease, ERK pathway, methylation, Notch signaling

## Abstract

The Notch signaling pathway plays critical role for determining cell fate by controlling proliferation, differentiation, and apoptosis. In the current study, we investigated the roles of the Notch signaling pathway in cigarette smoke (CS)-induced endothelial apoptosis in chronic obstructive pulmonary disease (COPD). We obtained surgical specimens from 10 patients with COPD and 10 control participants. Notch1, 2, and 4 express in endothelial cells, whereas Notch3 mainly localizes in smooth muscle cells. Compared with control groups, we found that the expression of Notch1, 3, and 4 decreased, as well as their target genes Hes1 and Hes2, while the expression of Notch2 and extracellular signal-regulated kinase (ERK)1/2 increased in COPD patients compared with controls, as well as in human pulmonary microvascular endothelial cells (HPMECs) when exposed to CS extract (CSE). Overexpression of Notch1 with N1ICD in HPMECs markedly alleviated the cell apoptosis induced by CSE. The ERK signaling pathway was significantly activated by CSE, which correlated with CSE-induced apoptosis. However, this activation can be abolished by N1ICD overexpression. Furthermore, treatment of PD98059 (ERK inhibitor) significantly alleviated CSE-induced apoptosis, as well as reduced the methylation of mitochondrial transcription factor A (mtTFA) promoter, which was correlated with CS-induced endothelial apoptosis. These results suggest that CS alters Notch signaling in pulmonary endothelial cells. Notch1 protects against CS-induced endothelial apoptosis in COPD through inhibiting the ERK pathway, while the ERK pathway further regulates the methylation of mtTFA promotor.

## INTRODUCTION

Chronic obstructive pulmonary disease (COPD) is a multicomponent disease characterized by a progressive and irreversible airflow obstruction and destruction of parenchyma, which is mainly caused by cigarette smoke (CS) (32). This disease is a major public health burden and is the third leading cause of mortality worldwide (25). However, the mechanism of pathogenesis has yet to be elucidated. Recent data suggested that abnormal apoptosis serves as a significant factor contributing to the destruction of pulmonary tissue in COPD (28). Studies from both animal model and human confirmed that endothelial apoptosis plays an important role in the pathogenesis of emphysema and COPD (4, 14, 26, 36, 37).

The Notch signaling pathway is a highly conserved cell signaling pathway, and it plays a critical role in “cell-cell” signaling communication (27). Up to now, four Notch receptors (Notch1–4), and five Notch ligands (Jag1, Jag2, and delta-like ligands DLL1, DLL3, and DLL4), which are transmembrane proteins, have been identified in mammals. By binding to the ligand proteins, Notch receptors were proteolytically cleaved twice by metalloproteinase and γ-secretase, respectively, releasing the Notch intracellular domain (NICD). Then, the NICD moves into the nucleus to regulate the expression of Notch target genes, e.g., Hes and Hey families (16). Notch signaling is functionally involved in regulating a number of cellular activities, including apoptosis (6, 21). Overexpression of Notch1 protects endothelial cell from apoptosis, while knockdown of Notch1 leads to increasing of apoptotic cells (5). A recent analysis has shown that in the airway epithelium of healthy smokers and smokers with COPD, several key components of the Notch signaling are downregulated compared with controls (30). However, the mechanism underlying the regulation of Notch1 signaling on endothelial apoptosis in COPD remains largely unknown.

Overexpression of constituted active form of Notch1 (N1ICD) suppressed Toll-like receptor-triggered extracellular signal-regulated kinase (ERK) activation in macrophages, suggesting that Notch1 should be an ERK regulator (39). The ERK pathway, a regulator of cellular processes, has been shown to increase following exposure to CS both in vivo and in vitro (12, 17). In addition, ERK activation plays an active role in CS-induced apoptosis (17). Thus we hypothesized that Notch1 is involved in CS-induced apoptosis through regulation of the ERK signaling pathway.

DNA methylation is a major epigenetic modification in human, which has profound effects on gene expression. Aberrant promoter methylation of multiple genes increases the risk of pulmonary emphysema and COPD (29). Our previous study has demonstrated that the involvement of mitochondrial transcription factor A (mtTFA) methylation in COPD and demethylation treatment might protect models from emphysema (18, 38). Recent studies suggested that the decrease of ERK1/2 leads to DNA demethylation, which confirmed the ERK pathway to be a DNA methylation regulator (11). However, there were no studies about whether the hypermethylation of mtTFA in COPD was associated with the ERK pathway or not.

In the present study, we assessed the role of Notch1 in CS-induced apoptosis both in vivo and in vitro. We detected the expression of Notch receptors and the level of endothelial apoptosis in lungs from patients with COPD as well as in primary human pulmonary microvascular endothelial cells (HPMECs) treated with CS extract (CSE). We also investigated the effect of Notch1 on CSE-induced HPMEC apoptosis by measuring the rate of apoptosis and the expression level of apoptotic proteins. Furthermore, we investigated the role of Notch1 in regulating the ERK pathway to further explore the underlying mechanism.

## MATERIALS AND METHODS

### 

#### Reagents.

Antibodies against Notch1, cleaved Notch1 (N1ICD), ERK1/2, phospho-ERK1/2 (p-ERK), cleaved caspase-3, and β-actin were purchased from Cell Signaling Technology: Notch1 (cat. no. 3608), N1ICD (cat. no. 4147), ERK1/2 (cat. no. 4695), p-ERK (cat. no. 4370), cleaved caspase-3 (cat. no. 9664), and β-actin (cat. no. 4970). Antibodies against Notch2, Notch3, and Hes1 were purchased from Abcam: Notch2 (ab8926), Notch3 (ab23426), and Hes1 (ab108937). Antibodies against Notch4 were purchased from Santa Cruz Biotechnology (sc5594). Antibodies against Hey2 were purchased from Proteintech (10597-1-AP). The ERK inhibitor PD98059 (P215) and γ-secretase inhibitor DAPT (*N*-[*N*-(3,5-difluorophenacetyl-l-alanyl)]-*S*-phenylglycinet-butyl ester; GSI-IX; D5942) were obtained from Sigma-Aldrich. The Annexin V + propidium iodide (PI) kit was purchased from BD Biosciences.

#### Lung tissue study.

This study was approved by the Institutional Ethics Committee of the Second Xiangya Hospital of Central South University. We recruited consecutive patients from June 2013 to April 2014. Human lung tissue from patients with COPD (*n* = 10) and controls (*n* = 10) was obtained from patients undergoing surgery and who gave informed consent. All the 20 patients were enrolled with primary lung cancer of stage 1 and had the surgery of pulmonary segmentectomy or lobectomy. All the patients were clinically stable for 4 wk without acute pulmonary infection, did not receive chemotherapy half a year before the study, and did not have obstructive atelectasis, metastasis, other pulmonary diseases, and severe diseases in other systems. The diagnosis of COPD was based on the global strategy for the diagnosis, management, and prevention of COPD published in 2013 (32). The lung tissue 5 cm away from tumor margin was used in our studies. The pathological examination confirmed that these samples presented lung structure without metastasis or inflammation.

#### Apoptosis assay.

Terminal deoxynucleotidyl transferase-mediated dUTP nick end labeling (TUNEL) assay was performed to measure the apoptosis of endothelial cells in medium-sized pulmonary blood vessels (50 mm < diameter < 150 mm) of COPD patients according to the manufacturer’s instructions.

#### Immunohistochemistry.

Paraffin-embedded sections were deparaffinized and rehydrated with xylene and ethanol. Antigens retrieval was performed via the microwave method with citrate buffer for 20 min. Endogenous peroxidase, avidine, and biotin were blocked by 3% hydrogen peroxide. Normal bovine serum (5%) was used for blocking. Following this, the samples were incubated overnight, at 4°C, with a rabbit polyclonal antibody, respectively (Notch1, 2, 3, 4 and ERK1/2, p-ERK1/2, 1:100; Hes1 and Hey2, 1:50). Following the incubation with peroxidase-conjugated secondary goat anti-rabbit IgG, the sections were stained with 3,3′-diaminobenzidine (DAB). Negative control was established without addition of the primary antibody. Sections were then scored by two independent observers in a blinded fashion (number of Notch1-, 2-, 3-, 4-, Hes1-, Hey2-, p-ERK1/2-, and t-ERK1/2-positive endothelial cells/100 endothelial cells per case) for Notch1, 2, 3, 4, Hes1, Hey2, p-ERK1/2, and t-ERK1/2 staining in medium-sized blood vessels.

#### Primary cells and cell culture.

HPMECs were purchased from ScienCells Research Laboratory (Carlsbad, CA) and were cultured in endothelial cell medium supplemented with 5% FBS, 1% endothelial cell growth factor, and penicillin (100 IU/ml)/streptomycin (50 µg/ml) in a humidified, 5% CO_2_ atmosphere at 37°C. Only cells at passages 3 to 4 were used in experiments.

#### Preparation of CSE.

CSE was prepared as previously described (36). Briefly, one nonfiltered Fu-Rong cigarette (tar: 12 mg/cigarette; nicotine: 1.1 mg; and carbon monoxide: 14 mg; China Tobacco Hunan Industrial., Changsha, China) was burned, and the smoke was passed through 25 ml of phosphate-buffered saline (PBS) via a vacuum pump. This product was supposed to be 100% CSE solution. The aqueous smoke extract was filtered through a 0.22-μm membrane filter to remove particles and bacteria, and the pH of CSE was adjusted to 7.2~7.4 before use.

#### Treatment by CSE or DAPT or PD98059.

HPMECs were cultured in increasing concentrations of CSE (0.0, 0.5, 1.0, and 2.5%) for 12 h and 1% CSE was selected for time-dependent experiments. Time duration tests included 0, 6, 12, and 24 h. For Notch1 inhibition, HPMECs were cultured with 1% CSE and DAPT for 12 h, with a pretreatment of DAPT for 24 h. For ERK blocking, we cocultured cells with 1% CSE and 10 μM PD98059 for 12 h. Cells were pretreated with PD98059 for 30 min before exposure to CSE.

#### Assessment of apoptosis of HPMECs.

The apoptosis was assessed using an Annexin V-FITC apoptosis kit (BD FACSCalibur, San Jose, CA). After treatment, cells were harvested and washed twice with ice-cold PBS and binding buffer and incubated with AnnexinV-FITC in dark for at least 15 min. Propidium iodide (PI) was added in the dark for 10 min at room temperature. The degree of apoptosis was determined as the percentage of cells positive for Annexin V and negative for PI by flow cytometry. Each experiment was performed in triplicate and repeated independently at least three times.

#### Generation of lentiviral vectors.

cDNA encoding a constitutively active form of Notch1 consisting of the intracellular domain (base pairs: 5,308-7,665; amino acids: 1,770-2,555; ICN) was subcloned into the multicloning site of the lentiviral vector pNL-IRES2-enhanced green fluorescent protein (EGFP) to generate pNL-N1ICD-EGFP. To generate lentiviral vectors, 10 μg pNL-N1ICD–EGFP were transfected along with 7.5 μg pHelper1.0 and 5 μg pHelper 2.0 into 293T cells in 15-cm plates at 70% confluence for 8 h. The medium was replaced with 10 ml DMEM supplemented with 10% FBS. After culturing for 48 to 72 h, the supernatants were harvested for virus titration.

#### Real-time PCR.

After extraction, RNA was reverse transcribed using the PrimeScript RT Reagent Kit (Takara) and assayed using SYBR Premix Ex TaqTM following the manufacturer’s instruction. All of the primers were obtained from Sangon (Shanghai, China). Real-time PCR was conducted on the Step-one ABI real-time RT-PCR system. All mRNA expression values were presented relative to GAPDH. The primers used for real-time PCR are as follows [forward (F) and reverse (R)]: Notch1 F: 5′-CGCCTTTGTGCTTCTGTTCTT-3′; Notch1 R: 5′-TCCCGCCGCTTCTTCTT-3′; Notch2 F: 5′-CCGTGTTGACTTCTGCTCTCT-3′; Notch2 R: 5′-TCCTACTACCCTTGGCATCCT-3′; Notch4 F: 5′-AAGATGTGGATGAGTGTGAGACC-3′; Notch4 R: 5′-ACAGGTGGCAGCAATACAGTC-3′; Hes1 F: 5′-CACTGATTTTGGATGCTCTGA-3′; Hes1 R: 5′-AGGTGCTTCACTGTCATTTCC-3′; Hey2 F: 5′-TCTCTCCACCTCTCTCTTGTCC-3′; Hey2 R: 5′-CAGGGTCGGTAAGGTTTATTGT-3′; and GAPDH F: 5′-AATGCATCCTGCACCACCAA-3′; GAPDH R: 5′-GTAGCCATATTCATTGTCATA-3′.

#### Western blotting.

A total of 50 μg of protein was separated on SDS-PAGE gel (Sigma) and transferred to PVDF membranes. The membrane was blocked with 5% nonfat dry milk in PBS containing 0.05% Tween for 1 h. Following blocking, these membranes were washed and incubated overnight, at 4°C, with a rabbit polyclonal antibody, respectively (Notch1, 2, 4, N1ICD, Hes1, Hey2, and cleaved caspase-3, 1:500; ERK1/2 and p-ERK1/2, 1:1,000). Next, the membrane was washed three times and incubated with horseradish peroxidase-labeled secondary antibody (anti-rabbit, 1:4,000; Santa Cruz Biotechnology) for 1 h at room temperature. After being washed, antigen-antibody complexes were analyzed by enhanced chemiluminescence detection system (ECL; BestBio Pharmacia Biotech) and protein levels were quantified with Quantity One Analysis Software (Bio-Rad Laboratories, Hercules, CA). β-Actin was probed to confirm equal protein loading and transfer.

#### Bisulfite sequencing PCR.

The methylation of mitochondrial transcription factor A (mtTFA) promoter was detected as previously described (18).

#### Statistical analysis.

All data are expressed as the means ± SD. A Student’s *t*-test was used to compare the difference between two groups. One-way ANOVA was used for multiple groups to analyze any differences between two groups. Tukey’s test or Dunnett’s T3 test was used for post hoc multiple comparisons according to the homogeneity test of variance. Linear correlation analysis was conducted using Pearson product moment correlations. *P* < 0.05 was considered to indicate a statistically significant difference.

## RESULTS

### 

#### The general information of the patients.

Characteristics of the two groups of study subjects are shown in Table 1. There were no differences in age or sex between the two groups (*P* > 0.05). Compared with the control group, both forced expiratory volume in 1 s (FEV_1_)/Pre (%) (percentage of FEV_1_ actual of FEV_1_ predicted) and FEV_1_/forced vital capacity (%) were lower in the COPD group (*P* < 0.05).

**Table 1.** Clinical data of the study subjects

	Control (*n* = 10)	COPD (*n* = 11)
Male/female (*n*)	7/3	9/2
Age, yr	60.7 ± 7.6	63.4 ± 8.4
Smoking index, pack yr	0	43.1 ± 18.0*
FEV_1_/Pre (%)	94.9 ± 9.9	60.6 ± 19.5*
FEV_1_/FVC (%)	76.3 ± 3.7	58.1 ± 10.2*

Values are presented as *n* or means ± SD. COPD, chronic obstructive pulmonary disease. FEV_1_, forced expiratory volume in 1 s; Pre (%), percentage of FEV_1_ actual of FEV_1_ predicted; FVC, forced vital capacity.

* *P* < 0.05 compared with nonsmoking control group.

#### Apoptosis in vascular endothelial cells in lung tissues of patients with COPD and controls.

**Fig. 1.**
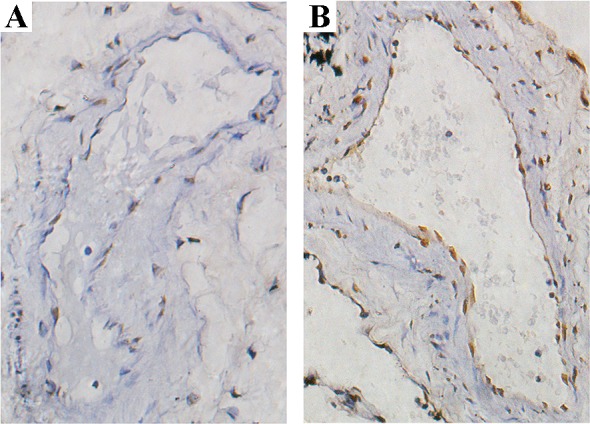
TUNEL detection of pulmonary vasculature. Positive cells were stained in yellow brown by TUNEL assay. Photomicrographs of TUNEL-stained lung tissue of control group (*A*) and chronic obstructive pulmonary disease group (*B*) (magnification: ×400 ).

The nuclei of TUNEL-positive cells were stained in yellow brown. The apoptotic index of pulmonary vascular endothelial cells in patients with COPD (18.47 ± 3.9%) was much higher than that in controls (6.5 ± 1.7%, *P* < 0.05; Fig. 1).

#### Expression of Notch receptors and p-ERK1/2 in lung tissue of patients with COPD and controls.

**Fig. 2.**
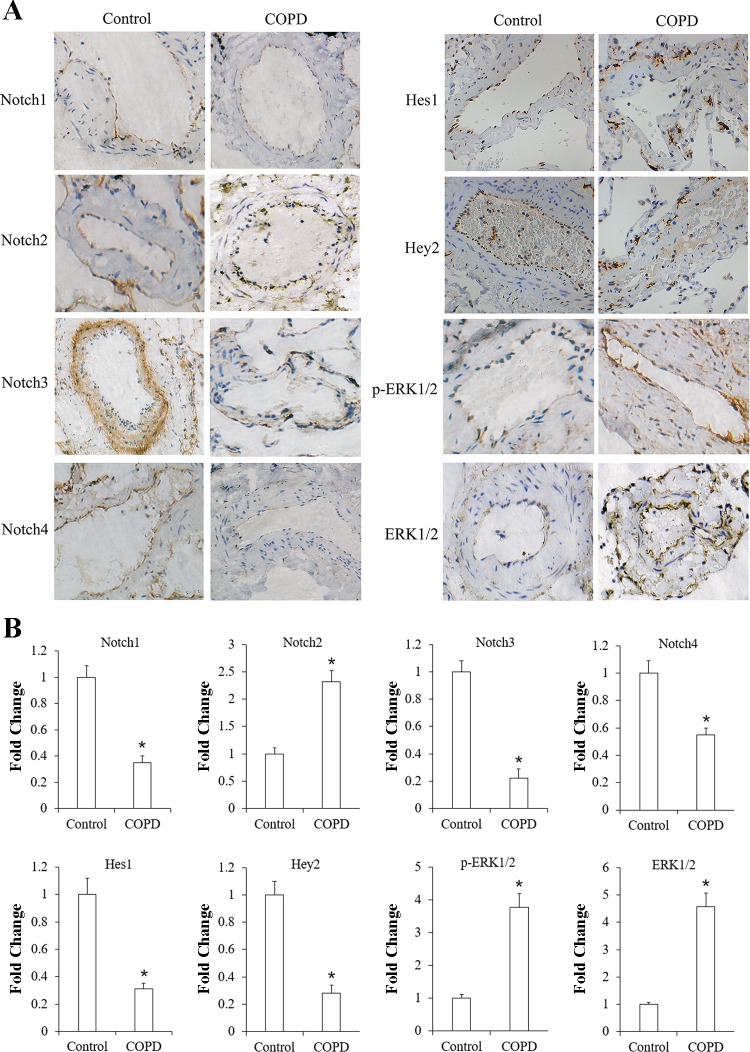
Immunohistochemical staining of Notch receptors and ERK1/2 in vascular endothelial cells of human lung tissues. Protein expression of Notch receptors, target genes Hes1 and Hey2, phosphorylated (p)-ERK1/2, and ERK1/2 was detected in the control group (*n* = 10) and chronic obstructive pulmonary disease (COPD) group (*n* = 10) (magnification: × 400). Protein expression was yellow brown in diaminobenzidine (DAB) staining (*A*). Positive cell indexes of Notch receptors, target genes Hes1 and Hey2, p-ERK1/2, and ERK1/2 in both groups (*B*). *P < 0.01 vs. control group.

We measured the protein expression level of the Notch receptors, the target genes Hes1 and Hey2, and p-ERK1/2 and t-ERK1/2 in lung tissue by immunohistochemistry. The receptors of Notch1, Notch2, and Notch4 could be detected in the endothelial cells. Compared with controls, the expression levels of Notch1 and Notch4 and the target gene Hes1 and Hey2 are lower in the patients with COPD. On the contrary, the expression of Notch2 was higher in COPD group as compared with the control group. Notch3 protein was mainly localized in smooth muscle cells of blood vessels, and its expression level in COPD patients was lower than that in controls. p-ERK1/2 and t-ERK1/2 expression in endothelial cells was higher in those patients with COPD as compared with the controls (Fig. 2).

#### CS-induced apoptosis in HPMECs.

**Fig. 3.**
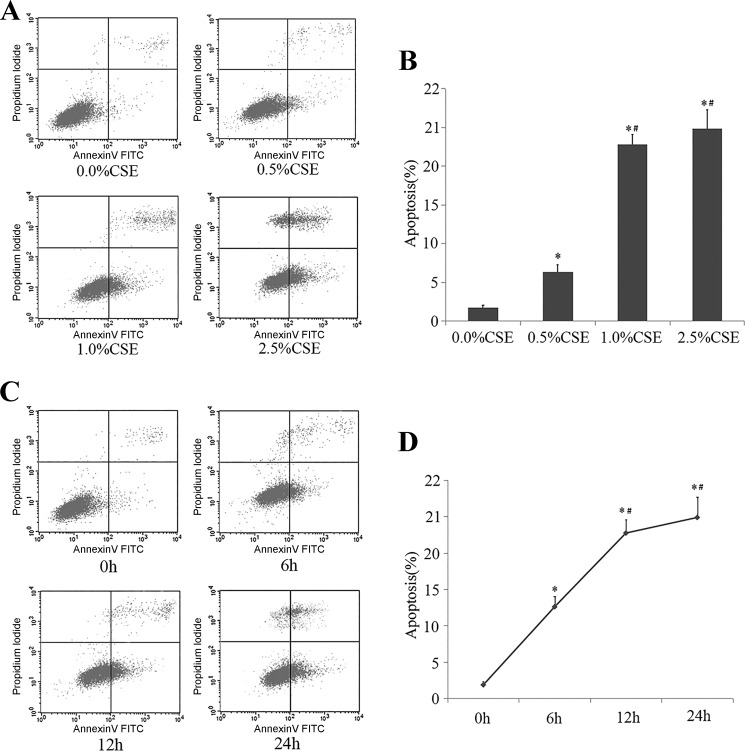
Flow cytometry analysis of human pulmonary microvascular endothelial cell (HPMEC) apoptosis induced by cigarette smoke extract (CSE). The Annexin V + propidium iodide staining shows the proportion of apoptotic cells treated by varied concentrations of CSE at 0.0, 0.5, 1.0, and 2.5% for 12 h (*A* and *B*), as well as under exposure to 1% CSE for 0, 6, 12, and 24 h (*C* and *D*). All experiments were performed independently at least 3 times. *P < 0.01 vs. 0.0% CSE group or 0h group; #*P* < 0.01 vs. 0.5% CSE-group or 6h group.

As shown in Fig. 3, FITC-Annexin V + PI assay showed that HPMEC apoptosis began to increase significantly when they were treated with 0.5% CSE compared with 0.0% CSE (*P* < 0.01) and further increased upon treatment with 1.0% CSE and 2.5% CSE (*P* < 0.01), suggesting a dose-dependent effect of CSE on HPMEC apoptosis. After incubation with 1% CSE, a time-related increase of CSE-induced apoptosis was observed from 6 h.

#### Modulation of Notch receptors and the target genes by CSE in HPMECs.

**Fig. 4.**
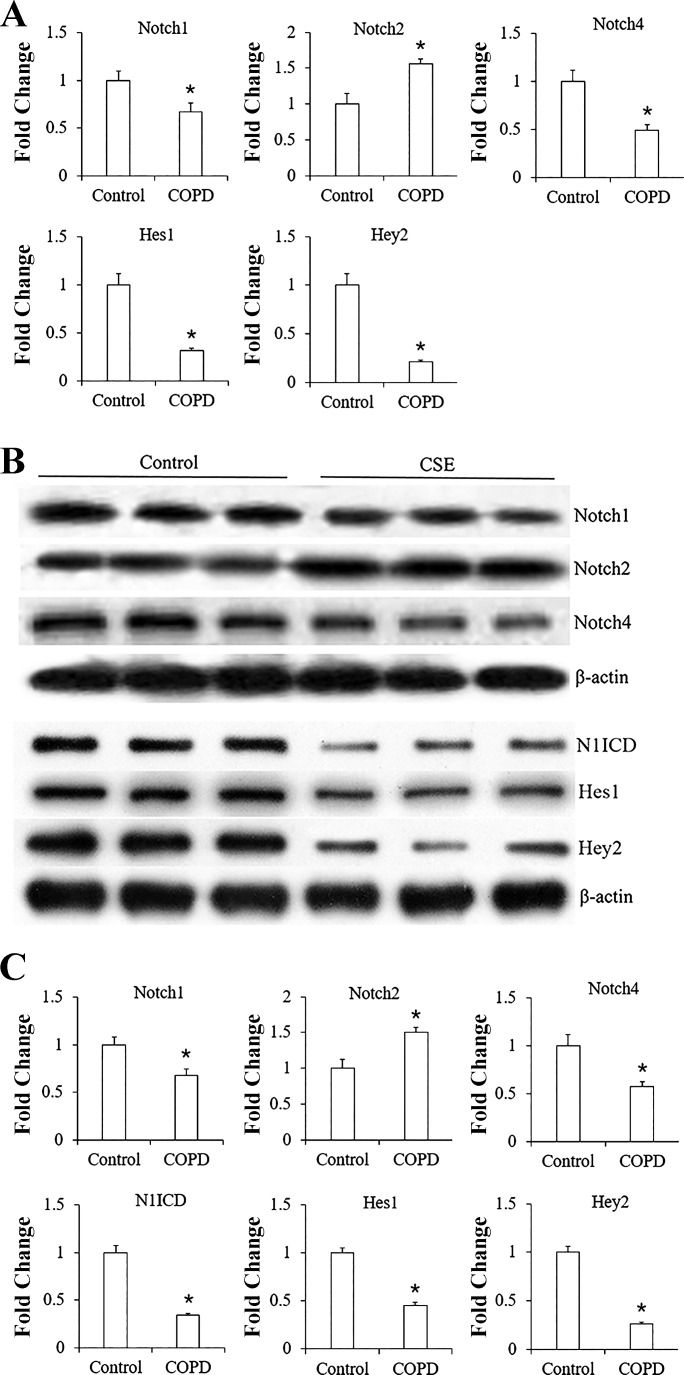
Expression of Notch receptors in human pulmonary microvascular endothelial cells (HPMECs) and modulation of Notch signaling by cigarette smoke extract (CSE). *A*: mRNA levels of Notch1, 2, 4 and the target genes Hes1 and Hey2 in HPMECs were normalized with respect to GAPDH. Expression of Notch1 and 4, Hes1 and Hey2 in the CSE group was significantly decreased, whereas Notch2 expression was increased. *P < 0.01 vs. control group. *B*: protein levels of Notch1, 2, 4, N1ICD, and target genes Hes1 and Hey2 in HPMECs. *C*: density of Notch1, 2, 4, N1ICD, Hes1, and Hey2 protein was normalized against β-actin to obtain a relative blot density. CSE decreased the expression of Notch1, 4, N1ICD, Hes1, and Hey2 and increased the expression of Notch2. *P < 0.01 vs. control group. All experiments were performed independently at least 3 times. COPD, chronic obstructive pulmonary disease.

mRNA expression of Notch receptors was detected in HPMECs by real-time PCR. A 12-h treatment with 1% CSE decreased the expression of Notch1 and Notch4, as well as the target gene Hes1 and Hey2, whereas it increased the mRNA level of Notch2 in HPMECs (Fig. 4*A*, *P* < 0.01 vs. controls).

Consistently, the protein levels of Notch1, Notch4, N1ICD, Hes1, and Hey2 were significantly decreased, whereas Notch2 increased in CSE-treated HPMECs in comparison with the controls (Fig. 4, *B* and *C*, *P* < 0.01).

#### Notch1 signaling protects HPMECs from CSE-induced apoptosis.

**Fig. 5.**
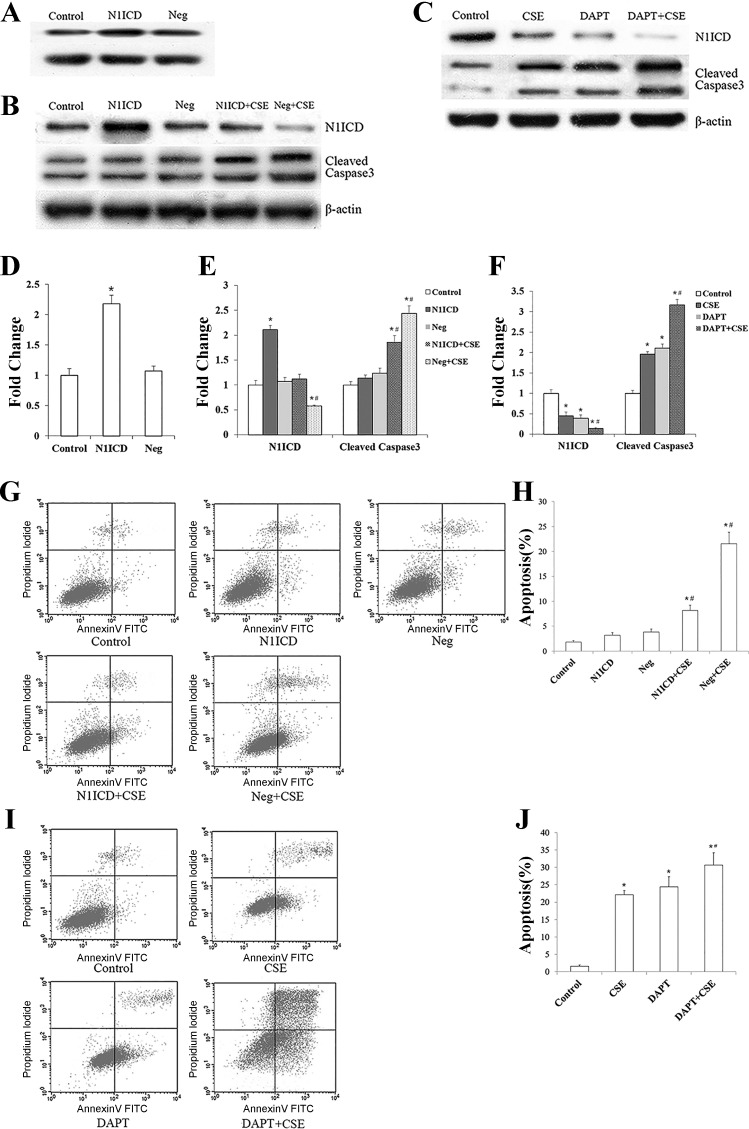
Effect of Notch1 overexpression on apoptosis of human pulmonary microvascular endothelial cells (HPMECs). Western blotting was used to verify whether N1ICD functions in the HPMECs (*A* and *D*) and to detect changes of N1ICD and cleaved caspase-3 after the overexpression of N1ICD with or without cigarette smoke extract (CSE; *B* and *E*). After treatment with DAPT, a novel γ-secretase inhibitor, protein levels of N1ICD and cleaved caspase-3 were detected by Western blotting with or without CSE (*C* and *F*). β-Actin was used as loading control. Annexin V + propidium iodide assay showing the proportion of apoptotic cells in each experimental group after N1ICD overexpression (*G* and *H*) or Notch1 inhibition (*I* and *J*). Neg, negative control. All experiments were performed independently at least 3 times. *P < 0.01 vs. control group; #*P* < 0.01 vs. N1ICD group or CSE group.

We used a gain-of-function approach to determine whether Notch1 activation can influence CSE-induced HPMEC apoptosis or not. We transfected these cells with a lentiviral vector coexpressing a flag-tagged N1ICD and used the EGFP (N1ICD group) or an EGFP vector as a negative control (Neg group). Then, the N1ICD- and Neg-transfected cells were exposed to 1% CSE for 12 h. N1ICD expression was detected by Western blot, which indicated that theN1ICD lentiviral vector properly functions in these cells (Fig. 5, *A*, *B*, *D*, and *E*; *P* < 0.01 vs. control).

We then studied the effects of Notch1 overexpression on the apoptosis of HPMECs. As shown in Fig. 5, *B* and *E*, there was no significant difference in the protein expression of cleaved caspase-3 in N1ICD group, Neg group, and control group (*P* > 0.05). After CSE exposure, the protein levels of cleaved caspase-3 in both N1ICD + CSE group and Neg + CSE group were increased (*P* < 0.01 vs. control). However, the increase in N1ICD + CSE group was not as great. We also analyzed the apoptosis rate of HPMECs by FITC-Annexin V + PI assay after N1ICD overexpression (Fig. 5, *G* and *H*). Consistently, the apoptosis rates were similar to those achieved with Western blot assay for the expression of cleaved caspase-3 protein.

To further confirm the protective role of Notch1 in CSE-induced endothelial apoptosis, DAPT, a novel γ-secretase inhibitor, which is required for proteolytic processing of Notch1 receptors, was used. As shown in Fig. 5, *C* and *F*, the protein level of N1ICD was reduced in CSE group as well as DAPT group and further reduced in DAPT + CSE group (*P* < 0.01). An increase of cleaved caspase-3 protein was detected in the CSE group, DAPT group, and CSE + DAPT group, and the cotreatment of CSE and DAPT showed the most significant increase of cleaved caspase-3 (*P* < 0.01). Similar to the change of cleaved caspase-3, the apoptosis rates detected by the FITC-Annexin V + PI assay (Fig. 5, *I* and *J*) were also increased in the CSE group and DAPT group and further increased in the CSE + DAPT group.

#### Notch1 inhibits CSE-induced endothelial cell apoptosis via ERK-dependent mechanisms.

**Fig. 6.**
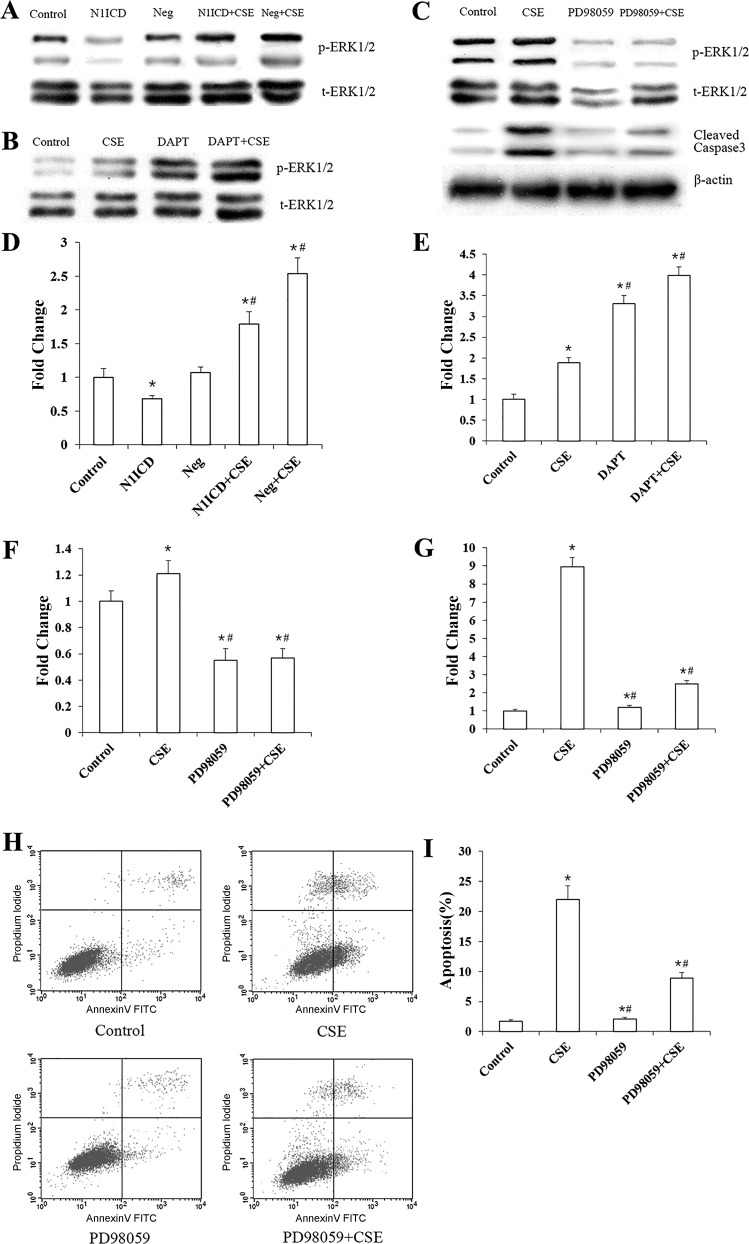
Notch1 overexpression inhibits cigarette smoke extract (CSE)-induced apoptosis via ERK1/2 pathway in human pulmonary microvascular endothelial cells (HPMECs). After transfection with N1ICD lentiviral vector, HPMECs were treated with CSE or left untreated. ERK1/2 protein levels in each experimental group were analyzed by Western blotting (*A* and *D*). After treatment with DAPT, protein levels of ERK1/2 were detected by Western blotting with or without CSE (*B* and *E*). HPMECs were pretreated with 10 μm PD98059 for 30 min and then stimulated with 1% CSE for 12 h or left untreated. ERK1/2 and cleaved caspase-3 protein levels in the control, CSE, PD98059, and PD98059 + CSE group were analyzed by Western blotting (*B*, *F*, and *G*). β-Actin was used as loading control. Levels of cell apoptosis in control, CSE, PD98059, and PD98059 + CSE experimental groups were also assessed by Annexin V + propidium iodide assay (*H* and *I*). Neg, negative control. All experiments were performed independently at least 3 times. *P < 0.01 vs. control group; #*P* < 0.01 vs. N1ICD group or CSE group.

We addressed whether Notch1 signaling could influence the ERK pathway in our pulmonary endothelial cell model. As shown in Fig. 6, *A* and *D*, compared with control group (*P* < 0.01), the expression level and activity of ERK1/2 in N1ICD group both decreased. The protein level of ERK1/2 in both N1ICD group and Neg group increased after CSE exposure (*P* < 0.01). However, the increase in N1ICD + CSE group was not as much as in Neg + CSE group. Then, we detected the inhibition effect of Notch1 on the ERK pathway (Fig. 6, *B* and *E*). The expression of ERK1/2 increased in the CSE group and DAPT group and further increased in DAPT + CSE group (*P* < 0.01).

Next, we detected the effect of the ERK pathway on endothelial cell apoptosis by inhibiting the ERK pathway with PD98059. As shown in Fig. 6, *C* and *F*, treatment of PD98059 significantly reduced the expression and activity of ERK1/2 (*P* < 0.05), indicating that PD98059 exerted an effective inhibition in the ERK pathway. The expression level of the cleaved caspase-3 increased in CSE-treated HPMECs (Fig. 6, *C* and *G*; *P* < 0.01), and PD98059 partially reversed the increase level of cleaved caspase-3 that was induced by CSE (*P* < 0.01). Consistently, compared with control group, the FITC-Annexin V + PI assay shows a significant increase in cell apoptosis in CSE group. Blockade of the activation of the ERK pathway effectively restored the CSE-induced cell apoptosis (Fig. 6, *H* and *I*).

#### Involvement of the ERK pathway in mtTFA methylation.

**Fig. 7.**
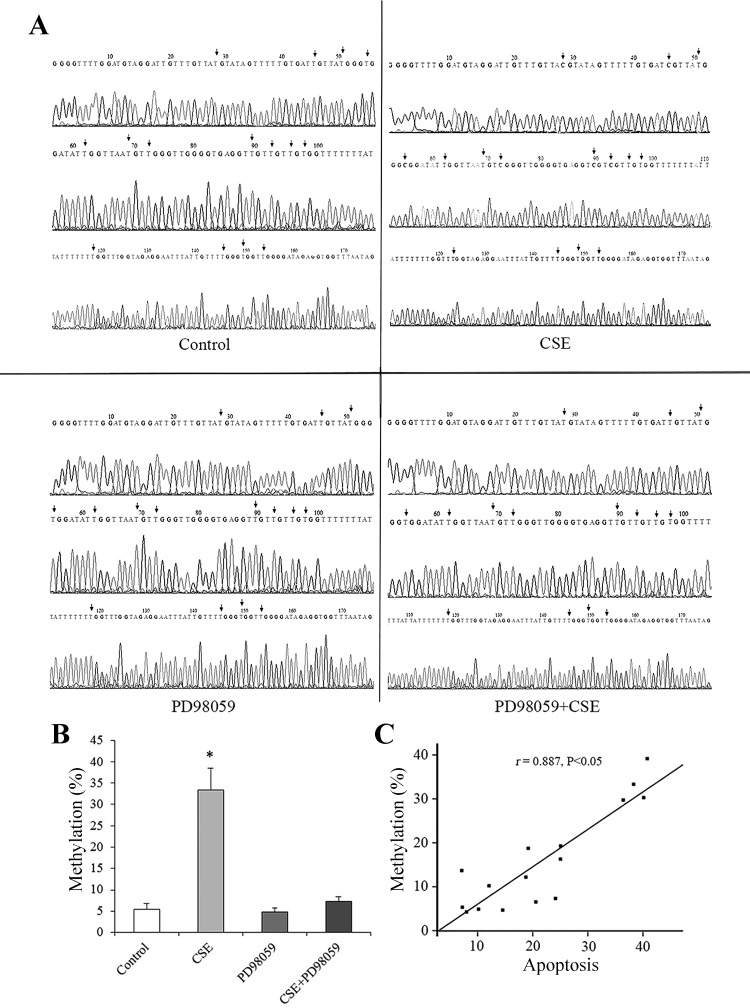
Effect of ERK1/2 on the methylation of mitochondrial transcription factor A (mtTFA) promoters and correlation analysis of mtTFA promotor methylation and apoptosis in human pulmonary microvascular endothelial cells (HPMECs). The percentage of methylated CpG dinucleotides in the mtTFA promoter was measured by bisulfite sequencing PCR in each experimental group (*A* and *B*). All experiments were performed independently at least 3 times. *P < 0.01 vs. control group. Scatterplot of correlation analysis of the level of mtTFA promoter methylation and apoptosis (*r* = 0.887, *P* < 0.01) in HPMECs (*C*). CSE, cigarette smoke extract.

Finally, we detected the role of the ERK pathway in DNA methylation. HPMECs were pretreated with PD98059 and then stimulated with CSE, and the mtTFA methylation was detected by bisulfite sequencing PCR and DNA sequence analysis. As shown in Fig. 7, compared with the controls, the mtTFA promoter methylation increased significantly in CSE group, which was restored by PD98059 treatment. More importantly, the methylation level of mtTFA promoter was positively correlated with CSE-induced HPMEC apoptosis (*r* = 0.887, *P* < 0.05).

## DISCUSSION

The present study showed that the Notch1, Notch2, and Notch4 receptors could be detected in the vascular endothelial cells, whereas the Notch3 protein was mainly localized in smooth muscle cells of blood vessels. The expression of Notch1 and Notch4 and the target gene Hes1 and Hey2 decreased obviously, while the Notch2 and ERK pathway enhanced in patients with COPD, as well as in CSE-treated endothelial cells in vitro. Overexpression of Notch1 with N1ICD protected the endothelial cell against the apoptosis that was induced by CSE through inactivation of the ERK pathway. Moreover, the ERK pathway was correlated with CSE-induced endothelial apoptosis through regulation of mtTFA promotor methylation. Taken together, our study suggests that Notch1 is involved in CS-induced endothelial apoptosis in COPD through regulation of the ERK pathway and the ERK pathway further regulates the methylation of mtTFA promotor. These data provide promising preclinical evidence for targeting COPD by combinatorial alteration of these two pathways.

Recent data have highlighted an association between CS, apoptosis, and the development of emphysema in COPD. Due to a decrease in cell maintenance factors, it has been proposed that epithelial and endothelial cell death may be responsible for the development of emphysema (18). Consistent with this notion, our previous studies have confirmed that CS exposure induces endothelial cell apoptosis and small vascular injury in both animal models and human lungs (4, 18, 37, 38). An increase in pulmonary endothelial cell apoptosis induced by CSE in vitro was also reported (4, 36). Correspondingly, the present in vitro study showed a dose- and time-dependent apoptosis of HPMECs when exposed to CSE. These results indicated that the endothelial apoptosis contributed to the pathogenesis of COPD.

Notch components are found to be extensively expressed during the initial stages of lung development, which may be crucial to regulate the respiratory epithelium and the vascular endothelium (19, 31). The expression of Notch receptors displays a selective cellular and tissue distribution. In the vasculature, both Notch1 and Notch4 are expressed in vascular endothelial cells, while Notch4 displays an almost exclusively endothelial expression pattern (10, 24). Notch3 is primarily expressed in adult arterial vascular smooth muscle cells. Consistent with previous observations, we found that Notch1, Notch2, and Notch4 receptors are all expressed in pulmonary vascular endothelial cells, while Notch3 was mainly localized in vascular smooth muscle cells.

Notch signaling is involved in cell fate determination from *Drosophila* to human, affecting the proliferation, differentiation, stem cell maintenance, and apoptosis of diverse cell types and the development of numerous organ systems (27). Notch1/4 have been reported to play a protective role in endothelial cell apoptosis, inhibiting of Notch1/4 leading to abundant apoptotic cell death (5, 23), whereas overexpression of Notch1/4 protected cells from apoptosis (5, 15). Notch2 is confirmed to have an opposite effect on Notch1/4 (24). Despite these findings, the role of Notch signaling in CS-induced apoptosis is still unclear. The present study demonstrated that Notch1 and Notch4 expression decreased both in vivo in COPD and in CSE-treated endothelial cells. Converse changes were observed in the expression of Notch2. These results implied a role of Notch signaling in the apoptosis of pulmonary endothelial cells and in the pathogenesis of COPD. Due to the similar biological effects of Notch1 and Notch4 on endothelial cell regulation and the opposite effect of Notch2 on the former two receptors, we selected Notch1 as a target gene to further study the role of Notch signaling in regulating endothelial apoptosis induced by CS. Based on our present study, it is compelling to speculate that Notch1 could qualify as one such molecular mediator and modulation of Notch1 signaling in the endothelium may be beneficial for patients with COPD. Using lentivirus-mediated overexpression, we found that overexpression of N1ICD dramatically decreased CSE-induced endothelial apoptosis. These data demonstrated that Notch1 was a protective factor against CSE-induced apoptosis in endothelial cells. However, the underlying mechanisms responsible for this are still poorly understood.

The ERK/MAPK signaling pathway regulates multiple biological processes including development, apoptosis, autophagy, oncogenesis, and inflammation (9). Several studies have described upregulation of the ERK pathway following CS exposure both in vivo and in vitro (12, 17). In agreement, our present results revealed that the expression and phosphorylation of ERK1/2 were increased in COPD patients and in endothelial cells treated with CSE. As two distinct signaling pathways, both Notch and ERK exert significant effect on cell apoptosis, and the two pathways may display some degree of cross talk. Recent evidence suggests that the inhibition of Notch1 signaling by the γ-secretase inhibitor promoted the expression and activation of the ERK pathway (39). Cai et al. (3) also found that blockade of Notch signaling led to enhanced phosphorylation of ERK. Consistent with these results, our present study demonstrates that overexpression of Notch1 with N1ICD transfection led to decreased expression and phosphorylation of ERK1/2, suggesting Notch1 to be a negative regulator of the ERK pathway.

Until now, the exact role of ERK in apoptosis is still controversial. Some studies suggested that ERK inhibition attenuates CSE-induced cell death and expression of apoptosis-related proteins, such as caspase-3 and caspase-8 (17), whereas others showed that ERK functions as a survival factor that can inhibit apoptosis (20). In the present study, we found that the expression of ERK1/2 was positively correlated with CSE-induced endothelial apoptosis and the inhibition of ERK by PD98059 effectively attenuates the cell apoptosis. Wada and Penninger (33) reported that MAPK signaling might either protect or enhance sensitivity to apoptosis depending on the cell type, stimuli, and the latency of the activation of MAPKs. It seems likely that in the case of CS stimulation, the activation of the ERK pathway was associated with enhanced endothelial apoptosis. Above all, our findings demonstrate that Notch1 signaling negatively regulates CS-induced endothelial apoptosis through regulation of the ERK pathway.

DNA methylation is a major epigenetic modification in human, which has profound effects on gene expression. Aberrant promoter methylation of multiple genes was associated with an increased risk for pulmonary emphysema and COPD (1, 29). Previous researches indicated that CS not only impacted the global epigenetic pattern but also induced the methylation of individual genes (2, 22). Recent research suggested that decreased ERK1/2 can lead to DNA demethylation through regulation of DNA methyltransferase 1, confirming the ERK pathway to be a DNA methylation regulator (11). Thus we speculated that the hypermethylation of DNA may be partially due to the activation of the ERK pathway.

mtTFA is mandatory for both transcription and maintenance of mitochondrial DNA. It has been shown that mtTFA plays an important role in regulating apoptosis. Disruption of the mtTFA gene in mice results in depletion of mtDNA, impairment of chain respiration, and increased cell apoptosis (34), while overexpression of mtTFA can ameliorate the decline in mtDNA copy number and apoptosis in transgenic mice (8). Our previous studies confirmed that the expression of mtTFA was decreased by CS, which was negatively related to the apoptosis. The decrease of mtTFA was closely related to the hypermethylated mtTFA promoter induced by CS (18, 38). Furthermore, demethylation therapy can effectively decrease the level of mtTFA methylation, increase the mtTFA expression, and reverse the endothelial apoptosis and emphysema induced by CS (38). These in vivo studies supported the role of methylation in endothelial apoptosis and in the development of COPD by finding the hypermethylated mtTFA promoter. Here, our in vitro study reconfirmed that CSE stimulation can induce the mtTFA promoter methylation, and the present research verified the hypothesis that blockade of the ERK pathway with PD98059 significantly reduced the level of mtTFA promoter methylation. In addition, the level of mtTFA promoter methylation was positively correlated with endothelial apoptosis, which has been confirmed by our previous studies (18, 38). These data give us an implication that the ERK pathway may be implicated in CS-induced endothelial apoptosis via mediating mtTFA promoter methylation.

In conclusion, our findings suggest that the expression of Notch1 and Notch4 is significantly decreased, whereas Notch2 is increased, in the pulmonary vascular endothelial cells in COPD patients. Overexpression of Notch1 shows a significant anti-apoptotic effect on endothelial cells via inhibiting the ERK pathway. Furthermore, the activation of the ERK pathway by CS may promote cell apoptosis via regulating mtTFA promotor methylation, which has been confirmed to play a role in CS-induced pulmonary endothelial apoptosis. These findings not only highlight the importance of the cross talk of Notch1 with ERK pathways but also suggest a new avenue of combination therapy for CS-related diseases, such as COPD. Regardless, our findings cast light on the protective effect of Notch1 signaling on endothelial apoptosis induced by CS, which may provide insight into the pathogenesis of COPD.

### 

#### Limitations of the study.

First, the present study elucidates a clear mechanism of regulation of CS-induced endothelial apoptosis by Notch1 signaling. We select Notch1 as a representative receptor to further study the role of Notch signaling in regulating endothelial apoptosis induced by CS. However, Notch2 and Notch4 may also function in this process. In our next study, we will explore the effect of overexpression or inhibition of the two receptors on endothelial apoptosis induced by CS and decide what role they play in the pathogenesis of COPD. Second, accumulating evidence supports the existence of important cross-talk between Notch and several other apoptosis-related pathways like ERK, phosphatidylinositol 3-kinase/Akt, and Wnt/β-catenin (7, 35, 39). The present study mainly demonstrates that the inhibition of Notch1 can promote endothelial apoptosis via the ERK pathway. It is important to further investigate whether Notch signaling can regulate CS-induced endothelial apoptosis through other signaling pathways. Third, numerous studies of preclinical animal models and cultured cells have demonstrated that CS exposure increases pulmonary endothelial barrier permeability and pulmonary endothelial cell apoptosis. After CS-induced injury to endothelial cells, components of smoke enter the circulation via a more permeable endothelial barrier (13), subsequently leading to circulating endothelial cell injury. Thus the endothelial injury of the pulmonary vasculature might be more obvious than other circulating endothelial. However, it is hard to distinguish pulmonary from aortic vasculature in the lung tissue samples. At present, it is universally acknowledged that CS can induce pulmonary endothelial apoptosis. Combined with the cell studies, we can draw the following conclusion that CS can induce pulmonary endothelial apoptosis.

## GRANTS

This study was supported by National Natural Science Foundation of China (Grants 81170036, 81270100, and 81370143); the Key Research and Development Program of Hunan Science and Technology Department (Grant 2015SK20403); and the National Key Clinical Specialty Construction Projects of China (Grant 2012-650).

## DISCLOSURES

No conflicts of interest, financial or otherwise, are declared by the authors.

## AUTHOR CONTRIBUTIONS

D.Z. and P.C. conceived and designed research; D.Z. and S. Cai performed experiments; D.Z., J.L., S.H., Q.L., J.J., S. Chen, Y.L., Y.C., and R.O. analyzed data; D.Z., J.L., S.H., Q.L., J.J., S. Chen, Y.L., Y.C., and R.O. interpreted results of experiments; D.Z. prepared figures; D.Z., J.L., S.H., Q.L., J.J., S. Chen, Y.L., Y.C., and R.O. drafted manuscript; D.Z., S. Cai, and P.C. edited and revised manuscript; D.Z., J.L., S. Cai, S.H., Q.L., J.J., S. Chen, Y.L., Y.C., P.C., and R.O. approved final version of manuscript.
